# Radiological Findings in Multidetector Computed Tomography (MDCT) of Hereditary and Sporadic Pulmonary Veno-Occlusive Disease: Certainties and Uncertainties

**DOI:** 10.3390/diagnostics11010141

**Published:** 2021-01-19

**Authors:** Marta Pérez Núñez, Sergio Alonso Charterina, Carmen Pérez-Olivares, Yolanda Revilla Ostolaza, Rafael Morales Ruiz, Ana Belén Enguita Valls, Jair Antonio Tenorio, Natalia Gallego Zazo, Alicia De Pablo Gafas, Pablo Lapunzina, Adriana Rodríguez Chaverri, Pilar Escribano Subías

**Affiliations:** 1 Servicio de Radiología, Unidad Multidisciplinar de Hipertensión Pulmonar, Hospital Universitario Doce de Octubre, 28041 Madrid, Spain; sealonsoch@gmail.com (S.A.C.); yolanda.revilla@salud.madrid.org (Y.R.O.); rmoralesr@salud.madrid.org (R.M.R.); 2 Centro de Referencia Nacional de Hipertensión Pulmonar Compleja, Spain and ERN-Lung-Pulmonary Hypertension) Referal Center, 28041 Madrid, Spain; carmenperezolivaresd@gmail.com (C.P.-O.); anabelen.enguita@salud.madrid.org (A.B.E.V.); alicia.pablosga@salud.madrid.org (A.D.P.G.); adrianarodriguezchaverri@gmail.com (A.R.C.); pilar.escribano.subias@gmail.com (P.E.S.); 3 Servicio de Cardiología, Unidad Multidisciplinar de Hipertensión Pulmonar, Hospital Universitario Doce de Octubre, 28041 Madrid, Spain; 4 Ciber-CV: Instituto de Investigación Sanitaria Hospital Doce de Octubre (imas12), 28041 Madrid, Spain; 5 Fundación Para la Investigación Biomédica del Hospital Universitario Doce de Octubre (FIBH12O), 28041, Madrid, Spain; 6 Servicio de Anatomía Patológica, Hospital Universitario Doce de Octubre, 28041 Madrid, Spain; 7 Institute of Medical and Molecular Genetics (INGEMM)-IdiPAZ, Hospital Universitario La Paz-UAM, Paseo de La Castellana, 261, 28046 Madrid, Spain; jairantonio.tenorio@gmail.com (J.A.T.); nataliagallegozazo@gmail.com (N.G.Z.); plapunzina@gmail.com (P.L.); 8 CIBERER: Centro de Investigación Biomédica en Red de Enfermedades Raras, ISCIII, Melchor Fernández Almagro, 3, 28029 Madrid, Spain; 9 ITHACA: European Reference Network on Rare Congenital Malformations and Rare Intellectual Disability, 1000 Brussels, Belgium; 10 Servicio de Neumología, Unidad de Trasplante Pulmonar, Hospital Universitario Doce de Octubre, 28041 Madrid, Spain

**Keywords:** pulmonary veno-occlusive disease, pulmonary hypertension, multidetector computed tomography (MDCT)

## Abstract

Pulmonary veno-occlusive disease (PVOD) is a very infrequent form of pulmonary arterial hypertension with an aggressive clinical course, poor response to specific vasodilator treatment, and low survival. Confirming a definitive diagnosis is essential to guide treatment and assess lung transplantation. However, in the absence of histological or genetic confirmation, the diagnosis is complex, requiring a clinical suspicion. Multidetector computed tomography (MDCT) is an essential part of the non-invasive diagnostic tools of PVOD. We retrospectively reviewed the MDCT findings from a consecutive series of 25 patients diagnosed with PVOD, 9 with the sporadic form and 16 with the hereditary form of the disease. The presence and extent of typical findings of the diagnostic triad were assessed in all patients (ground glass parenchymal involvement, septal lines, and lymphadenopathy). In our series, 92% of patients showed at least two of the radiological findings described as typical of the disease. All patients presented at least one typical radiological characteristic. The incidence of radiological findings considered typical is very high, however was not associated with greater hemodynamic severity nor to the development of acute lung edema. No significant differences were found between the two groups. A poorly expressive MDCT does not exclude the disease.

## 1. Introduction

Pulmonary veno-occlusive disease (PVOD) is a very infrequent form of pulmonary arterial hypertension (PAH) whose incidence is estimated in 0.2–0.5 cases per million inhabitants/year, although the true incidence is suspected to be higher [1]. This disease, together with pulmonary capillary hemangiomatosis (PCH), is part of group 1.6 of the clinical classification of pulmonary hypertension [2]. From the hemodynamic point of view, it corresponds to a precapillary PH, defined by a mean pulmonary arterial pressure (PAPm) ≥25 mm Hg, a pulmonary arterial wedge pressure or pulmonary capillary pressure (PCP) ≤15 mm Hg and pulmonary vascular resistance (PVR) ≥3 units Wood [3].

PVOD is an uncommon disease that may present in a wide range of ages, although most cases are diagnosed in the fifth and sixth decade of life. In hereditary cases, the disease usually presents at younger ages [4]. It does not show a gender predilection and no clear risk factors have been identified, although there is an association with certain conditions such as previous chemotherapy (mainly alkylating agents), HIV infection, tobacco, and scleroderma, among others [5].

The most important advance in the diagnosis of PVOD is related to genetic analysis. Biallelic pathogenic variants in the EIF2AK4 gene have been found to be associated with PVOD and PCH, in an autosomal recessive pattern of inheritance with apparently full penetrance [6]. Pathogenic variants in this gene have not been described in patients with idiopathic arterial hypertension (IPAH) [7], confirming that variants in this gene definitely cause PVOD. Biallelic pathogenic variants in the EIF2AK4 gene have been identified in 100% of patients with inherited PVOD. However, it is only present in a low percentage of patients with sporadic PVOD (5–9%) [8], so its absence does not discard the disease. Furthermore, earlier investigations described two phenotypes of hereditary PVOD in Romani ethnicity with important prognostic differences depending on their tolerance to pulmonary vasodilators and pulmonary pathological findings [9,10].

The clinical presentation of patients with PVOD does not differ significantly from IPAH. In both entities, dyspnea on exertion, palpitations, and syncope represents the most frequent clinical manifestations. However, it is important to differentiate between these two entities due to the different therapeutic management, worse prognosis, and lower survival of patients with PVOD. Median survival from the onset of symptoms is approximately two years, although disease progression in children may be faster. The therapeutic options are limited and, so far, the only curative option offered is lung transplantation [5,11].

The pathological characteristic of PVOD is the lesion of the postcapillary pulmonary vessels, with disorganized hypertrophy of the smooth muscle and deposit of collagen matrix in the small pulmonary veins and capillaries, phenomena of thrombosis with occlusion of the vascular lumen, and arterialization of the vein walls. The postcapillary obstruction characteristic of this disease is the cause of the increase in hydrostatic pressure and the development of acute pulmonary edema (APE) that occurs after the administration of arterial vasodilators (used in the treatment of PAH) and of areas of pulmonary hemorrhage that can be seen in histological specimens [12].

PVOD represents a diagnostic challenge due to the impossibility of obtaining a definitive histological diagnosis, since in most cases, lung biopsy carries a high risk of bleeding, therefore it is contraindicated [13]. However, the diagnosis can be made with high probability by combining clinical suspicion, physical examination, respiratory function test, and radiological findings [3]. Respiratory function tests show normal spirometry with decreased PaO2 and a severe decrease in diffusing capacity for carbon monoxide (DLCO) [5] in most patients. A clinical finding of great interest when it appears is the development of acute lung edema after the start of vasodilator treatment for PAH, a finding that is considered a diagnostic criterion for PVOD [7].

Among the diagnostic imaging tests, lung scintigraphy is not helpful in the diagnosis of PVOD although it maintains a leading role to distinguish chronic thomboembolic pulmonary hypertension (CTEPH) from PAH [1]. MDCT plays a relevant role in the PVOD diagnosis. Typical findings that indicate PVOD are septal lines, ground glass opacities, and mediastinal lymphadenopathy. The association of these three findings has been published to have a specificity of 100% for PVOD in PAH cases, and a sensitivity of 66%. Furthermore, its presence may be closely related to the risk of pulmonary edema associated with the treatment of PAH [3].

Since it is a very rare disease, the published series of patients are small and there are few studies on the radiological manifestations of this disease. Given the importance that MDCT plays in the diagnosis, the objective of this work is to analyze the frequency of radiological findings considered typical of this pathology in a consecutive series of 25 patients with diagnosis of PVOD in our center, Reference Unit of the National Health System (CSUR) for Pulmonary Hypertension.

We have analyzed the possible existence of significant radiological differences between sporadic and hereditary forms of the disease. In the group of patients with sporadic disease, where genetic diagnosis is not possible, the clinical-radiological correlation acquires vital importance, hence our interest in finding clues in the differential diagnosis.

## 2. Material and Methods

We retrospectively reviewed the MDCTs (Philips Brilliance 64-slice CT, Philips, Netherlands) of 25 patients with a confirmed diagnosis of PVOD (see below). In all cases, the MDCT study included axial images of the lung parenchyma with slice thickness ≤2 mm. All studies were analyzed by four thoracic radiologists with at least 4 years of experience in the pulmonary hypertension unit of our hospital. Differences were resolved by consensus.

The interobserver variability in the results of MDCT was analyzed between radiologists with more and less experience.

The diagnosis of PVOD was established by either (1) histological analysis of the explanted lungs (in patients who underwent lung transplantation or who died), (2) by the presence of the biallelic pathogenic variants in the EIF2AK4 gene (by Sanger sequencing or custom massive parallel sequencing panel), or (3) by developing APE after PAH treatment with vasodilator drugs [13].

The demographic, hemodynamic data, and pulmonary function data were obtained from the data base of the Spanish Registry of Pulmonary Hypertension (REHAP) [14] as well as their informed consent for the inclusion in the study.

### 2.1. Histological Analysis

Hematoxylin-eosin-saffron staining was used in all histological samples to characterize pulmonary vascular abnormalities. All the studied specimens revealed typical pathological characteristics of PVOD.

### 2.2. Radiological Analysis

In all studies, the presence and extent of findings considered typical of the disease were analyzed [15]: (1) Pulmonary ground-glass opacities (defined as areas of increased pulmonary attenuation with preservation of visualization of the underlying vessels), (2) presence of septal lines (defined as linear opacities between the pulmonary lobules and that translate the thickening of the septa interlobular), and (3) mediastinal and hilar adenopathies of pathological size (short axis > 1 cm) [16].

Ground-glass lung involvement was classified as centrilobular nodular (small nodules with poorly defined contours occupying the center of the secondary lobule) and geographic (patchy areas with well-defined contours by the limits of the secondary lobules, as these are diffusely affected).

To analyze the severity of lung involvement, both lungs were divided into three regions (upper, middle, and lower) and classified by a graduation in which 0 was considered normal (no involvement), 1 when the involvement was present in one region (mild involvement), 2 when observed in two regions (moderate involvement) and 3 when findings were found in all three regions (severe involvement). This grading was performed independently for ground glass involvement and for the presence of septal lines.

In all cases the presence of pleural and pericardial effusion, the diameter of the pulmonary artery trunk (measured in an axial plane at its bifurcation), the diameter of the ascending thoracic aorta (measured in the same plane), the relationship between the diameter of the pulmonary artery trunk and the diameter of the ascending aorta, and the relationship between the transverse diameter of both ventricles. Pathological findings considered were a pulmonary artery trunk diameter ≥29 mm, a pulmonary artery trunk diameter/ascending aorta ratio >1 in patients under 50 years of age, and a right ventricle/left ventricle (RV/LV) ratio >0.9.

The presence of two or three typical findings was considered a positive radiological diagnosis for PVOD.

### 2.3. Statistical Analysis

The data have been expressed by means of median and interquantile range for continuous variables and absolute and relative frequency for qualitative variables. Fisher’s exact test was used and to obtain the value of p in continuous variables we used the non-parametric U-Mann–Whitney test. Survival analysis was carried out using the Kaplan–Meier method, defining the value of the event as death or lung transplantation. Statistical analysis was performed with STATA IC/14 (Stata 14.2, Company Statacorp, College Station, TX, USA). The level of statistical significance was set at *p* < 0.05.

Cohen-Kappa coefficient was calculated to analyze the interobserver variability estimating the agreement according to the Landis and Koch assessment [17].

## 3. Results

### 3.1. Demographic and Clinical Characteristics

The mean age at the time of diagnosis was 38 years (Table 1). Patients with the hereditary form had an earlier age of presentation (32 vs. 59 years, *p* < 0.0001) and a lower rate of comorbidities and cardiovascular risk factors. No predominance by gender was found (14 men and 11 women). None of the included patients had a history of occupational exposure to trichloroethylene or other organic solvents. The most frequent symptom was dyspnea, and the majority were in functional class III (64%) or IV (24%) of the New York Heart Association (NYHA). It should be noted that all patients had a diffusing capacity for carbon monoxide (DLCO) less than 50%.

Hemodynamically, all patients in the series had severe precapillary pulmonary hypertension, with mPAP of 49 mm Hg, normal PCP (10 mm Hg), and elevated PVR (9.87 UW).

Of the 25 patients studied, 16 (64%) were diagnosed with hereditary PVOD because they were carriers of biallelic pathogenic variants in the EIF2AK4 gene. Of these 16 patients, 11 patients had a family history and the founder mutation [c.3344C > T (p.P1115L)] was found in all of them. Of the five remaining patients with no family history (previously classified as sporadic PVOD until the genetic study was carried out), three belonged to the gypsy ethnic group and were carriers of the same homozygous founder mutation, the remaining two were carriers of a different mutation than founder, one in homozygosity and the other in heterozygosity (none with a family history of consanguinity).

**Figure 1:**
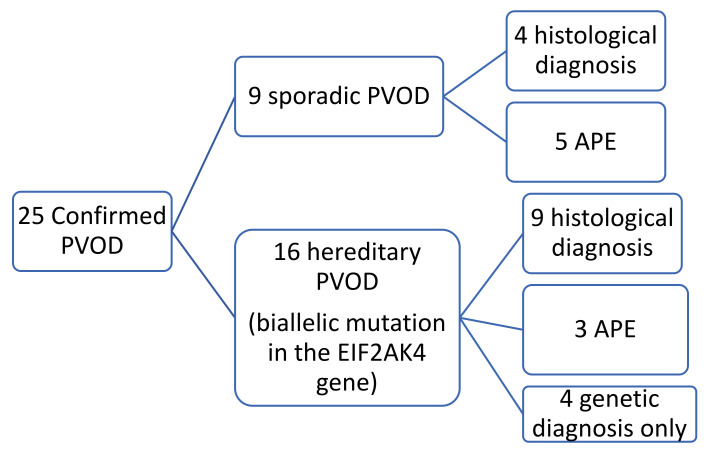
Diagram of included patients. PVOD: Pulmonary veno-occlusive disease. APE: Development of acute pulmonary edema after vasodilator treatment.

**Figure 2:**
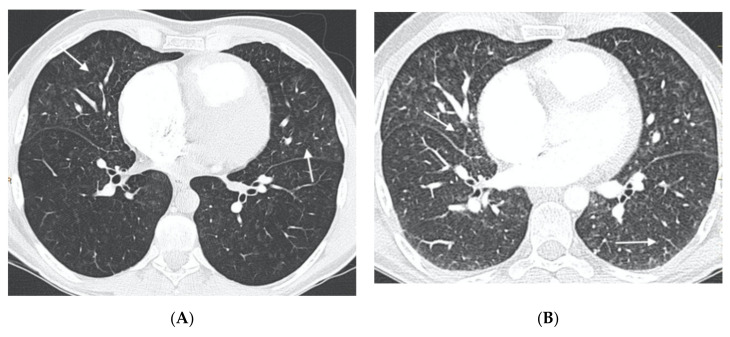
Forty-six-year-old man referred from another center with a diagnosis of idiopathic PAH. (**A**) MDCT on admission: parenchymal involvement with faint centrilobular nodules in ground glass (arrows); (**B**) MDCT after initiation of vasodilator treatment. Radiological worsening with increased ground glass involvement and the appearance of septal lines (arrows) in relation to pulmonary edema. The patient was finally diagnosed with hereditary PVOD.

In nine of these patients, the histological specimen was also available (in eight after double lung transplantation and in one by autopsy). Nine patients were diagnosed with sporadic PVOD, in four cases confirmed by pathological anatomy of the lung (two after double lung transplantation and two by necropsy) and the remaining five cases for developing APE after treatment with pulmonary vasodilator drugs (Figure 1 and Figure 2).

The mean transplant-free survival was 2.5 years, with a trend towards a worse prognosis in the subgroup of patients with sporadic disease (1.5 vs. 3.2 years, *p* = 0.2). Association between the survival and hemodynamic severity was not observed. However, the development of APE with vasodilator treatment was related to lower survival (Hazard Ratio 4, *p* = 0.01).

### 3.2. Radiological Characteristics

All the MDCT studies were performed at the diagnosis of the disease without pulmonary vasodilator treatment, with a median of 60 days prior to the first catheterization.

The majority of patients in both groups showed the typical signs of pulmonary hypertension (increased caliber of the pulmonary artery trunk ≥29 mm and/or a pulmonary artery trunk diameter/aorta ratio >1). Although both the frequency of this finding and the mean diameter of the pulmonary artery trunk were greater in the sporadic group, the differences were not statistically significant. All patients (except one in the hereditary group) showed signs of right overload with a RV/LV ratio >0.9. No significant radiological differences were observed between the two groups (Table 2).

Septal lines and mediastinal adenopathies were the most common findings in the sporadic group (100%). In the group with the hereditary form, the most frequent findings were ground glass involvement (94%) and the presence of septal lines (87%). In both groups, ground glass involvement was slightly more frequent in centrilobular distribution than in geographical distribution (Table 3).

The extent of parenchymal involvement, although it was greater in the hereditary form, does not present statistically significant differences except in the case of the centrilobular ground-glass extension, which is not relevant given the small number of patients (Table 4).

**Figure 3:**
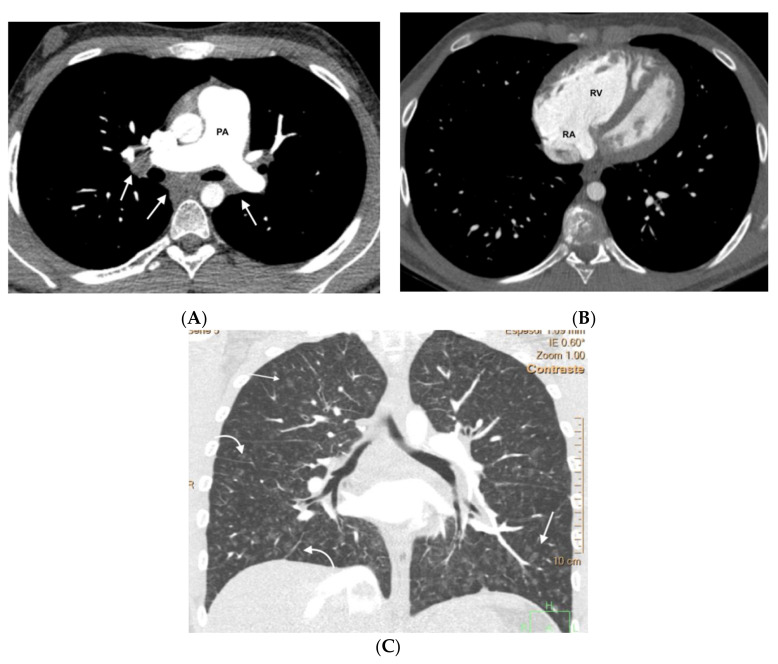
Fifteen-year-old male with hereditary PVOD and three typical findings of PVOD in MDCT. Radiological signs of pulmonary hypertension: (**A**) Dilation of the pulmonary artery (PA) trunk and (**B**) Dilation of the right heart chambers with RV/LV ratio >0.9, hypertrophy of the right ventricular wall, and inversion of the interventricular septum; (**C**) Extensive involvement in centrilobular nodular ground glass (arrow) and septal lines (curved arrow). Mediastinal lymphadenopathy in subcarinal and bilateral hilar locations (arrows in (**A**)). PA: pulmonary artery. RA: right atrium. RV: right ventricle.

**Figure 4:**
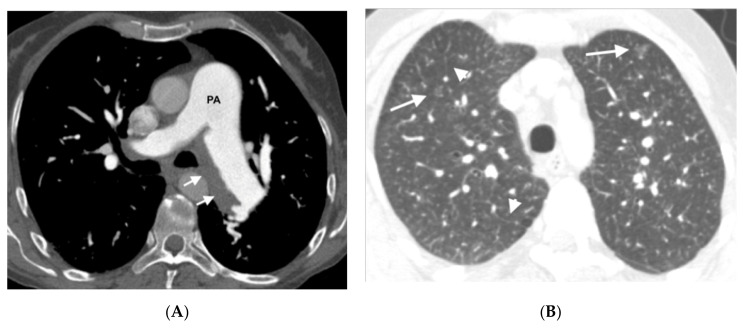

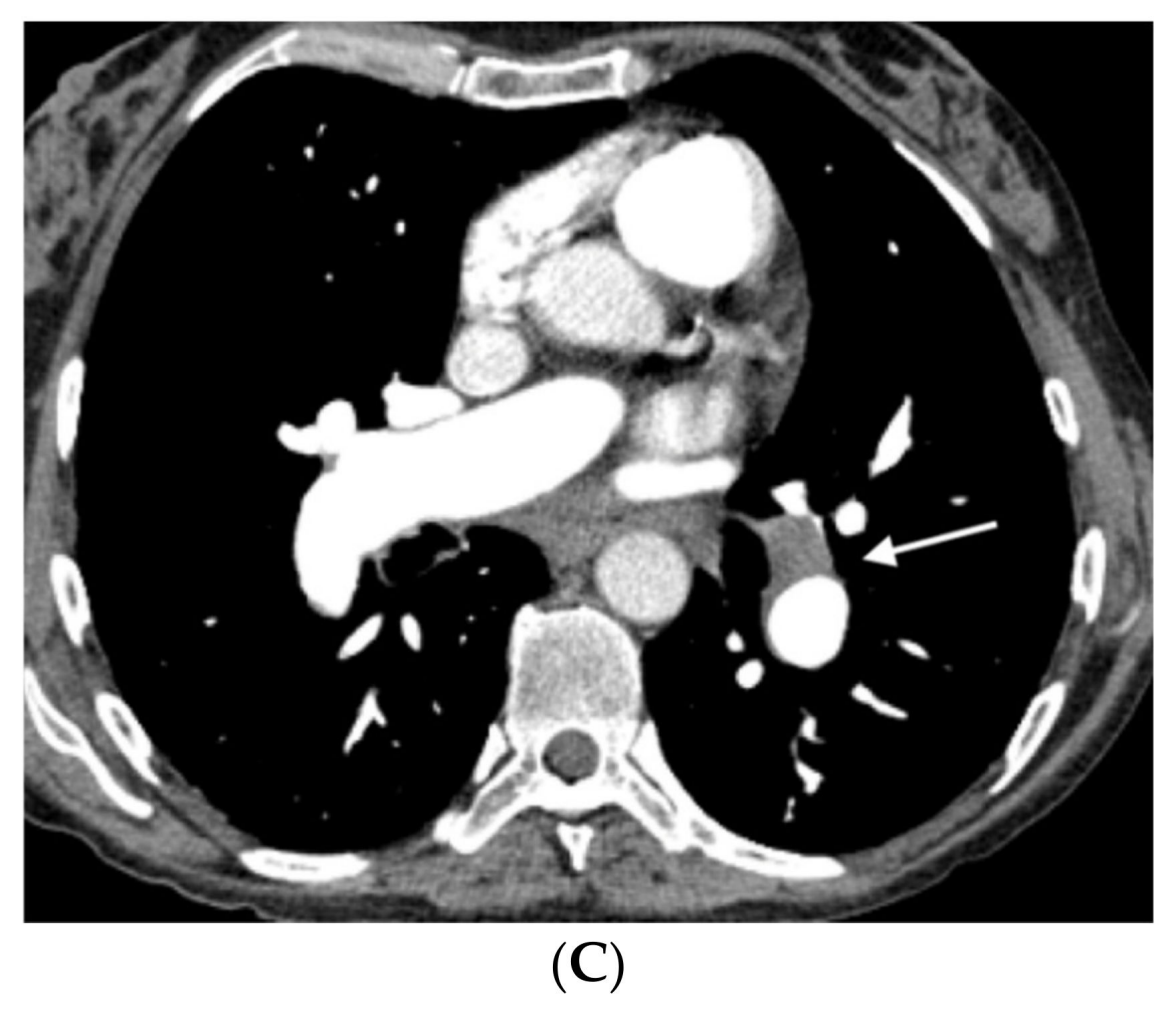
Fifty-five-year-old woman with sporadic PVOD histologically diagnosed after double lung transplantation. The patient had three typical MDCT findings and in situ thrombus in the left main pulmonary artery (normal ventilation-perfusion scintigraphy excluded CTEPH). (**A**) Dilation of the pulmonary artery (PA) trunk with thrombus in situ in the left main pulmonary artery (arrows); (**B**) ground-glass centrilobular nodules in both upper lobes (arrows) and septal lines (short arrows); (**C**) left hilar adenopathy (arrow). PA: pulmonary artery.

**Figure 5:**
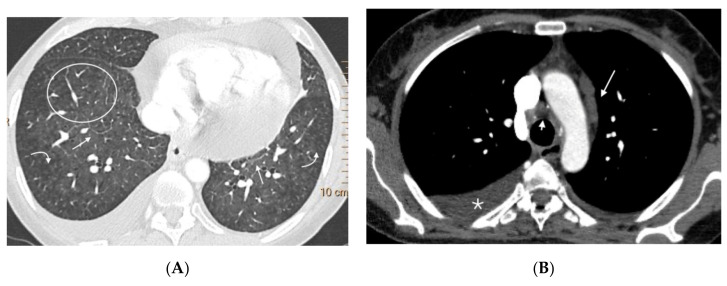
Forty-seven-year-old woman with sporadic PVOD and three typical findings. (**A**) Ground glass parenchymal involvement with central geographic distribution (circle) and peripheral centrilobular (curved arrows). Septal lines in basal regions of both lungs (straight arrows); (**B**) mediastinal lymphadenopathy in the lower right paratracheal location (short arrow) and prevascular (arrow). Right pleural effusion (asterisk).

**Figure 6:**
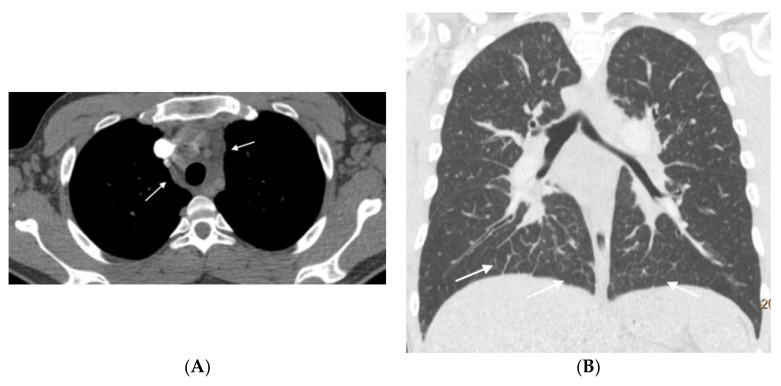
Nineteen-year-old male with hereditary PVOD and two typical findings. (**A**) Prevascular and right paratracheal lymphadenopathy (arrows); (**B**) coronal image. Septal lines in both lower lobes (arrows). There is no ground glass parenchymal involvement.

**Figure 7:**
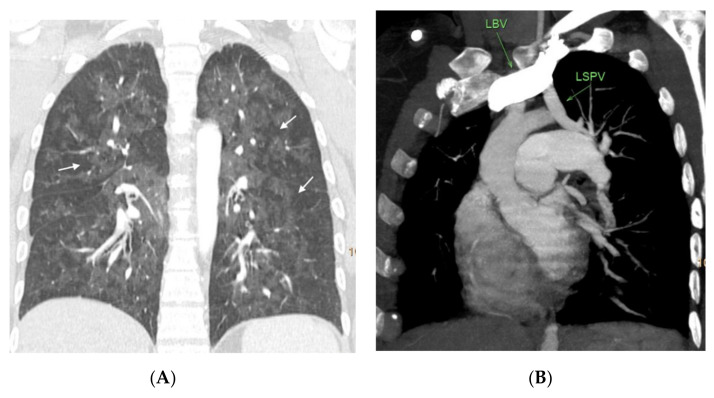
Thirty-three-year-old woman with hereditary PVOD with one radiological finding and partial anomalous pulmonary venous drainage. (**A**) Coronal reconstruction of the lung parenchyma. Extensive ground glass involvement showing geographic distribution (arrows), without evidence of septal lines; (**B**) oblique multiplanar reconstruction showing the abnormal drainage of the left superior pulmonary vein in the left brachiocephalic venous trunk. LSPV = left superior pulmonary vein. LBV = left brachiocephalic trunk.

In the group of patients with hereditary disease, 69% (11 patients) had the three typical findings and 19% (3 patients) had two findings. The remaining patients showed only a radiological finding. These two patients nevertheless presented hemodynamic severity and tolerated the specific vasodilator treatment without developing APE. In the group of patients with sporadic disease, 67% (6 patients) showed the three typical findings and 33% (3 patients) showed two findings (Figure 3, Figure 4, Figure 5, Figure 6 and Figure 7). No patients in the sporadic group had a low suspicion study (<1 finding) (Table 5). The presence of a greater number of typical findings was not associated with greater hemodynamic severity (*p* = 0.91), nor to the development of APE (*p* = 0.89).

Both pleural effusion and pericardial effusion were found more frequently in the sporadic group (38% and 67%, respectively) compared to the group of hereditary patients (7% and 29%, respectively), although the difference was not statistically significant.

Considering a diagnosis of MDCT positive for PVOD when two or three of the typical findings were present, the sensitivity of MDCT in our series was 88% in the group of patients with hereditary disease and 100% in the group of patients with sporadic disease. The kappa coefficient of interobserver agreement calculated between the two radiologists with the most and least experience was 0.65.

## 4. Discussion

PVOD is a rare subtype of PAH; it is difficult to diagnose and has poor prognosis, with a mean transplant-free survival of two years [9]. Establishing proper diagnosis is essential, due to therapeutic implications. Although the histological analysis provides the definitive diagnosis, lung biopsy is contraindicated due to the high risk of complications, with MDCT being a fundamental tool.

To the best of our knowledge, this work provides the largest series published to date of patients with confirmed PVOD who have been studied using MDCT. The series collected in the literature with confirmed PVOD are smaller, the largest with 20 patients and the smallest with 4 patients [15,18,19,20].

Our series includes 25 patients, 16 with the disease confirmed by genetic study and 9 with the sporadic form confirmed by histology (4 patients) or developing APE after vasodilator treatment (5 patients). According to previously published data, we observed a high incidence of typical findings (68% had three findings, and 24% showed two), and all had at least one typical finding. Therefore, MDCT findings can support the diagnosis of this entity.

Our study has also shown good interobserver agreement (kappa 0.65) in the reading of radiological images. This topic has not been previously analyzed but that reinforces the role of MDCT in the diagnosis of PVOD.

Resten et al. [18] reported for the first time statistically significant differences in thoracic MDCT findings when a group of 15 patients with histologically confirmed PVOD were compared to a group of 15 patients with IPAH. The key data for the radiological diagnosis of PVOD were ground-glass parenchymal opacities, the presence of septal lines, and enlarged mediastinal lymphadenopathy (short axis > 1 cm). Montani et al. analyzed 20 patients with confirmed PVOD and compared them with 13 patients with IPAH. The authors concluded that MDCT is highly suggestive of PVOD when >2 findings cited as keys in the diagnosis are found (present in >75% of patients in their series) [15].

In our series, 92% had at least two of the typical radiological findings and only 8% had a single radiological sign. These findings confirm MDCT as a key element in non-invasive diagnosis together with a marked decrease in DLCO [13]. However, their scarcity does not exclude the disease; in fact, in our series, two of the patients with hereditary PVOD showed only one radiological finding.

On the other hand, we have not come across radiological findings that allow us to select which patients will have a good response to specific vasodilator treatment or predict the development of APE, aspects that have not been previously studied in the literature.

Globally, septal lines were the most frequent finding in both groups (92%), as in previously reported series [8,15,18,19,20]. It was especially noted that 100% of patients in the sporadic group had visible septal lines and mediastinal adenopathies.

The presence of ground glass opacities is a common feature in PVOD (87% in our series), although this finding was not uncommon in patients with IPAH (33%) in the series by Resten et al. The presence of ground glass with a centrilobular distribution has been classically related to congestion at the alveolar level, and with an increase in blood volume at the capillary level, being considered more specific for PVOD than ground glass with a geographical distribution, more typical of IPAH [8]. In our study, centrilobular distribution was more frequent than geographical distribution in both hereditary and sporadic PVOD.

The prevalence of mediastinal lymphadenopathy is high in patients with PVOD (84% in our series), being less common in patients with IPAH (0–27%) [7,14,17], an entity with which we must establish the main differential diagnosis. Although they can also be observed in other groups of pulmonary hypertension (group 2: secondary to left heart disease and group 4: CTEPH), these groups differ from other complementary tests (cardiac catheterization and scintigraphy).

In 2014, the presence of a biallelic pathogenic variants in the EIF2AK4 gene were associated with the development of PVOD and PCH with a penetrance of 100% [9]. From the clinical point of view, they are younger patients, without associated cardiovascular risk factors, but with the same hemodynamic severity and mortality as sporadic PVOD [8,9]. In our study, a comparison between hereditary and sporadic PVOD was established without having observed significant differences in radiological characteristics. Although a greater extension of ground glass has been detected in hereditary PVOD, the percentage of diagnostic studies has been higher in sporadic PVOD, in contrast to the study by Hadinnapola et al. [8], which demonstrated a greater severity of the typical signs in patients with hereditary PVOD. Furthermore, the two patients with less radiological expressiveness (only one finding) corresponded to two patients with hereditary PVOD. This could suggest that active genetic screening in relatives allows an earlier diagnosis of the disease, which would lead to less radiological expressiveness in this subgroup of patients. However, the two patients with only one radiological typical finding had severe pulmonary hypertension at diagnosis (PVR 12 and 11.3 uW, respectively), a common feature being that both tolerated pulmonary vasodilator treatment including systemic prostacyclins. This finding could suggest that patients with lower radiological expressiveness would have a better tolerance to pulmonary vasodilator treatment [9].

Our study, however, has some limitations. First, although our series presents the largest number of published cases, it is still a small number, given the low incidence of the disease. Because of this, the ability to observe statistically significant differences based on subgroups is limited. Second, it is a retrospective and observational study in which the definitive diagnosis was already known prior to the radiological reading, which may have introduced a bias in the reading of the images. Third, there could be a selection bias in the patients with aggressive evolution, since all had a certain diagnosis of PVOD, either histological or genetic, 40% required transplantation, and the mean transplant-free survival was 2.5 years. So, there could be more aggressive forms of the disease and with greater radiological expressiveness.

In conclusion, the incidence of radiological findings considered typical of PVOD is very high, both in the sporadic and in the hereditary forms of the disease. No radiological criteria have been found that allow differentiating both forms, although the results suggest that in sporadic PVOD a higher radiological expression could be observed. Thoracic MDCT is an excellent diagnostic method, with little interobserver variability and reasonably safe for the patient, so we recommend its systematic use in patients in whom there is clinical suspicion of PVOD.

## Figures and Tables

**Table 1:** Demographics and clinical characteristics. FC: Functional class. DLCO: Diffusing capacity of the lung for carbon monoxide. mPAP: Mean pulmonary artery pressure. CO: Cardiac output; PAWP: Pulmonary arterial wedge pressure; PVR: Pulmonary vascular resistance; WU: Wood units; ProBNP: N-Terminal pro brain natriuretic peptide.

	Total (*n* = 25)	Hereditary (*n* = 16)	Sporadic (*n* = 9)	*p* Value
Age, years	38 (30–55)	32 (19–38)	59 (55–65)	<0.001
Sex: Male (%)	14 (56)	8 (50)	6 (67)	0.68
Smoking (%)	11 (44)	3 (19)	8 (89)	<0.01
Hypertension (%)	2 (8)	0 (0)	2 (22)	0.04
Diabetes (%)	3 (12)	1 (6)	2 (22)	0.53
Hypercholesterolemia (%)	5 (20)	1 (6)	4 (44)	0.08
Coronary disease (%)	2 (8)	0 (0)	2 (22)	0.12
FC, NYHA III-IV (%)	22 (88)	13 (81)	9 (100)	0.28
DLCO, %	32 (29–39)	32 (26–36)	36 (32–45)	0.13
6MWT, meters	240 (180–430)	310 (210–466)	210 (170–255)	0.2
mPAP, mm Hg	49 (39–63)	46 (39–62)	52 (44–66)	0.51
CO, l/min	4.05 (3.68–4.9)	4.09 (3.74–4.95)	3.90 (3.64–4.5)	0.51
PAWP, mm Hg	10 (8–11)	8.5 (6–11)	10 (10–11)	0.23
PVR, WU	9.87 (7.4–13)	8.84 (6.20–13.3)	11 (8–13)	0.4
ProBNP pg/mL	822 (89–2180)	364 (37–1382)	2180 (998–4600)	0.01
Lung transplant (%)	10 (40)	8 (50)	2 (23)	0.23
Survival free of transplant, years (SD)	2.5 (3.44)	3.2 (3.9)	1.5 (2)	0.2

**Table 2:** Frequency of radiological signs of pulmonary hypertension in patients with hereditary and sporadic PVOD. PA: Pulmonary artery; AA: Ascending aorta; RV: Right ventricle; LV: Left ventricle.

Radiological Finding	Total (*n* = 25)	Hereditary (*n* = 16)	Sporadic (*n* = 9)	*p* Value
PA dilation (>29 mm)	22 (92%)	13 (81%)	9 (100%)	0.4571
Diameter average PA (mm)	35 (32–40)	34 (31–38)	40 (35–40)	0.0371
PA/AA > 1	21 (87%)	13 (81%)	8 (89%)	0.9456
RV/LV > 0.9	24 (96%)	15 (94%)	9 (100%)	0.7659

**Table 3:** Frequency of radiological findings considered typical of PVOD in the hereditary and sporadic groups.

Radiological Finding	Total (*n* = 25)	Hereditary (*n* = 16)	Sporadic (*n* = 9)	*p* Value
Global ground glass	21 (87%)	15 (93%)	6 (66%)	0.1162
Centrilobular ground glass	9 (37%)	6 (37%)	3 (33%)	0.99
Geographic ground glass	4 (17%)	3 (19%)	1 (11%)	0.98
Centrilobular and geographic ground glass	8 (33%)	6 (37%)	2 (22%)	0.661
Septal lines	23 (92%)	14 (87%)	9 (100%)	0.7354
Mediastinal lymphadenopathy	21 (84%)	12 (75%)	9 (100%)	0.2854

**Table 4:** Frequency of radiological findings considered typical of PVOD (ground glass and septal lines) by degree of extension.

Radiological Finding	Total (*n* = 25)	Hereditary (*n* = 16)	Sporadic (*n* = 9)	*p* Value
Centrilobular ground glass extension	9 (37%)	6 (37%)	3 (33%)	0.99
Severe involvement	6	6	0	
Moderate involvement	1	0	1	0.0111
Mild involvement	2	0	2	
Geographic ground glass extension	4 (17%)	3 (19%)	1 (11%)	0.98
Severe impairment	2	2	0	
Moderate involvement	1	1	0	0.1353
Mild involvement	1	0	1	
Centrilobular and geographic ground glass extension *	8 (32%)	6 (37%)	2 (22%)	0.661
Severe involvement	7	5	2	
Moderate involvement	0	0	0	0.99
Mild involvement	1	1	0	
Septal lines	23 (92%)	14 (87%)	9 (100%)	0.7354
Severe involvement	9	5	4	0.8214
Moderate involvement	10	8	2	0.3495
Mild involvement	4	1	3	0.2283

* presence of ground glass combined to varying degrees in all lung fields.

**Table 5:** Frequency of radiological findings considered typical of PVOD in the hereditary and sporadic groups.

Typical Radiological Findings	Total (*n* = 25)	Hereditary (*n* = 16)	Sporadic (*n* = 9)	*p* Value
1	2 (8%)	2 (12%)	0	
2	6 (24%)	3 (19%)	3 (33%)	0.4406
3	17 (68%)	11 (69%)	6 (67%)	
